# Stair walking effects on feelings of energy and fatigue: Is 4-min enough for benefits?

**DOI:** 10.3389/fpsyg.2022.895446

**Published:** 2022-08-18

**Authors:** Kaitlyn E. Carmichael, Patrick J. O’Connor, Jennifer L. Gay

**Affiliations:** ^1^Department of Kinesiology, University of Georgia, Athens, GA, United States; ^2^Department of Health Promotion and Behavior, University of Georgia, Athens, GA, United States

**Keywords:** exercise, mental energy, mental fatigue, perceived exertion, stair climbing

## Abstract

**Purpose:**

Even low intensity exercise bouts of at least 15 min can improve feelings of energy (FOE) and reduce systolic blood pressure. However, little is known about the psychological outcomes of briefer exercise bouts, particularly for modes of exercise that are more intense than level walking, and readily available to many working adults. This study assessed the effects of a 4-min bout of stair walking on FOE and feelings of fatigue (FOF).

**Methods:**

Thirty-six young adult participants were randomized to either stair walking or seated control groups. All participants walked on level-ground from a laboratory to a nearby stairwell (~90 s) and were seated for 4 min before beginning their experimental condition. Stair-walking participants walked up and down one flight of 16 stairs at their own pace for 4 min, while control participants remained seated during that time. Participants walked back to the laboratory for post-condition assessments. Measures of blood pressure, heart rate, rated perceived exertion (RPE), and the intensity of feelings of mental energy, mental fatigue, physical energy, and physical fatigue were assessed pre-and post-condition. Separate one-way ANOVAs were conducted on change scores for all variables.

**Results:**

The stair climbing group experienced significant increases in heart rate [*F*(1,34) = 13.167, *p* < 0.001] and RPE [*F*(1,34) = 93.844, *p* < 0.001] that were not observed in the seated control group. Four minutes of self-paced stair climbing resulted in small changes and non-significant differences within and between groups in blood pressure as well as FOE and FOF.

**Conclusion:**

Although a 4-min self-paced exercise bout can convey short-term physiological health benefits, a 4-min bout of self-paced indoor stair walking in a stairwell was insufficient to lower blood pressure or change subjective FOE and fatigue in a sample that exhibited better than typical FOE and FOF at the pre-test.

## Introduction

Feelings of energy (FOE) and feelings of fatigue (FOF) are related but distinct psychological mood states that wax and wane each day (O’Connor, 2004). Multiple factors can influence these feeling states, including environmental factors such as light and temperature (te Kulve et al., 2017), individual characteristics and behaviors such as personality (Armon and Shirom, 2011) and sleep patterns (Tkachenko and Dinges, 2018) as well as many illnesses (Matura et al., 2018), prolonged mental activities (Smith et al., 2019), and depressant and stimulant drug use (Tomlinson et al., 2018). For example, a single dose of 200 mg caffeine appears to be the most reliable way to increases FOE and decrease FOF (Maridakis et al., 2009) while mental fatigue does not appear to reduce total running distance during small-sided soccer training games (Clemente et al., 2021).

A single bout of exercise is among the most reliable ways to temporarily increase FOE. A meta-analytic review of 16 experiments that measured both FOE and FOF and tested a total of 678 participants found that a single bout of exercise increased FOE compared to controls in 91% of the effect sizes calculated, and the standardized mean effect was 0.47 (Loy et al., 2013). Exercise-induced reductions in FOF were less consistent and were more likely to occur after low-to-moderate intensity exercise longer than 20 min. These experiments predominantly tested college students completing moderate intensity, cycling or strength training of 20–40 min duration.

There is a paucity of research testing the impact of short duration exercise bouts of 15 min or less on FOE and FOF and we are aware of three studies. One study found that four 30-s all-out cycling sprints followed by 4-min of active recovery increased FOE and decreased FOF (Monroe et al., 2016). A second found that 10-min of low-to-moderate intensity stair walking increased FOE and did not change FOF (Randolph and O’Connor, 2017). A third study, which did not measure FOF, found that six 5-min bouts of moderate-intensity walking every hour across a 6-h workday resulted in 16% higher FOE compared to uninterrupted sitting (Bergouignan et al., 2016). Whether a single shorter bout of stair walking also increases FOE is unknown. If so, this could be especially useful for sedentary office workers who often have limited time for breaks and easy access to stairs. This type of brief break in sedentariness at work, which would be more vigorous than level walking, could have multiple psychological and metabolic health benefits (Gay et al., 2018; Wolff et al., 2021).

The purpose of the experiment described here was to examine the influence of a single 4-min bout of stair walking on FOE and FOF. Four-minutes was selected as the dose because it shows promise for improving physiological outcome (Gay et al., 2021). Based on the studies summarized above, including the meta-analysis (Loy et al., 2013), it was hypothesized that FOE would increase compared to both a pre-walk baseline and a seated control condition and that FOF would be unchanged. A few prior investigations of acute exercise on FOE and FOF have reported data for each individual participant (e.g., Ward-Ritacco et al., 2016); however, no prior studies involving an exercise duration of less than 15-min appears to have reported data for each individual. Data for each individual are reported here in an attempt to better document individual differences in FOE and FOF responses to a brief bout of stair walking.

## Materials and methods

All study procedures were approved by the University of Georgia Institutional Review Board prior to recruitment and data collection (IRB #00001296). This study is part of a broader research project. Some study methods have been described previously (Gay et al., 2021).

### Participants

Participants were recruited through listserv emails and verbal announcements in university classes. Study participation was incentivized with extra credit or a 10-dollar gift card. Eligibility criteria included: (1) at least 18 years of age, (2) no contraindications to exercise as assessed by the Physical Activity Readiness Questionnaire (Thomas et al., 1992), and (3) no reported constraints to stair walking. Forty-six participants (mean age = 21.11, SE = 0.19, 78% female) were randomized to one of three groups: stair walking (*n* = 18); seated stairwell control (*n* = 18); or seated laboratory control (*n* = 10) using an online randomization scheme. A power calculation using a within measures correlation of 0.92 across trials and an alpha error of 0.05 revealed statistical power of 0.80 to detect an interaction effect size of 0.39 (D’Amico et al., 2001). Descriptive characteristics of participants are reported in Table 1.

**Table 1:** Participant characteristics by group (M ± SD).

	Seated laboratory (*n* = 10)	Seated stairwell (*n* = 18)	Stair walking (*n* = 18)
Age	21.30 ± 0.95	21.56 ± 1.62	20.56 ± 0.78^*^
Height (m)	1.67 ± 0.08	1.72 ± 0.09	1.72 ± 0.09
Weight (kg)	71.44 ± 13.73	68.95 ± 12.67	69.36 ± 14.33
BMI (kg/m^2^)	25.73 ± 4.80	23.17 ± 3.27	23.25 ± 3.50
Ascents/Descents	–	–	17.48 ± 2.52

* Significant differences (p < 0.05) compared to the seated stairwell group.

### Procedures

**Figure 1:**
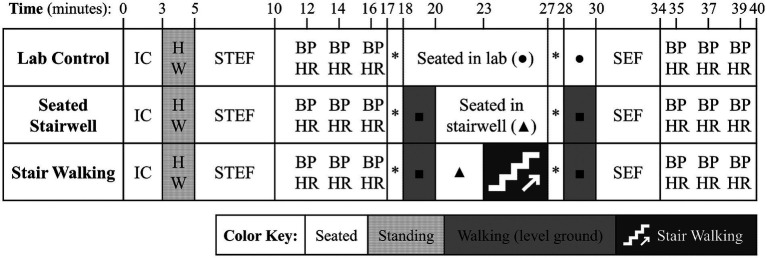
Experimental protocol. Symbols and abbreviations: *, measure of perceived exertion; 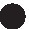, seated in lab; 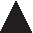, seated in stairwell; 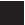, walking to and from stairwell, and 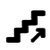, stair walking; BP, blood pressure; H, height; HR, heart rate; IC, informed consent; SEF, state energy and fatigue (post-experiment); STEF, state and trait energy and fatigue (pre-experiment); W, weight.

Figure 1 displays a timeline of the experimental protocol. At the beginning of the lab visit, participants were informed of all procedures and provided written consent. The researcher measured participants’ height and weight with a beam-balance scale and stadiometer. Next, participants were seated and completed measures of trait and state feelings of mental and physical energy and fatigue. After participants were seated for at least 5 min, blood pressure and heart rate were measured. The blood pressure cuff was placed on the left arm which was supported by a table. Participants were instructed to sit with both feet flat on the floor and breathe normally throughout the measurement. Blood pressure and heart rate were taken 3 times, with 2 min separating measurements. Then, participants in either the stair walking or stairwell control group were escorted by the researcher to the nearest stairwell. These participants entered from the ground floor of the stairwell and were asked to sit in a chair while researchers reviewed additional procedures including perceived exertion instructions. Participants in the laboratory control group did not walk at all and remained seated in the lab, matched for the same amount of time required for all procedures in the experimental group.

Participants in the stair walking group were instructed to continuously walk up and down the stairs at a self-selected pace for the duration of 4 min. The 4-min duration was selected as a novel duration that has demonstrated physiological benefits (Gay et al., 2021), and is of a vigorous intensity and long enough stimulus to potential activate energy and fatigue brain circuits (Stahl, 2002). Two taped lines were placed at the bottom and top of the flight of stairs to indicate the location of “turn around” points. Participants in the seated control condition remained in the chair for the same duration. At the end of the 4-min, participants were asked to report their perceived exertion and then escorted back to the lab. On return, participants were immediately seated. All groups completed post-measures of state mental and physical energy and fatigue. Finally, blood pressure and heart rate were measured a second time, using the same procedures described above.

### Measures

#### Blood pressure and heart rate

Blood pressure was taken manually using a stethoscope (Mabis Legacy Sprague Rapport) and aneroid sphygmomanometer (Mabis Legacy Professional). Average values of the three measurements were used as the criterion measure.

#### Perceived exertion

After being given standardized instructions, participants were asked to rate their perceived exertion during the experimental trial using a 15-point scale (Borg, 1998). This perceived exertion (RPE) scale ranges from 6 (no exertion at all) to 20 (maximal exertion) and displays strong psychometric properties as a measure of exercise intensity (Borg, 1998).

#### State and trait energy and fatigue questionnaire

Participants were asked questions pertaining to both their usual (trait) and current (state) intensity of FOE, vigor and pep as well as FOF, exhaustion, and being worn out. These descriptors are used in relation to participants’ perceived ability to perform physical or mental activities, resulting in subscales of physical energy, physical fatigue, mental energy, and mental fatigue. The trait scale uses a Likert-scale focused on frequency and ranging from 0 (never) to 4 (always), while the state scale is measured using a visual analogue scale focused on the intensity of feelings and ranging from 0 (no feelings) to 100 (strongest feelings ever felt). Subscale item scores are summed such that trait and state values range from 0 to 12 and 0 to 300, respectively. The STEF has been used in dozens of published studies (e.g., Loy and O’Connor, 2016; Ward-Ritacco et al., 2016; Frederick et al., 2022). Details about the STEF, along with normative data and psychometric supportive evidence, are available in a manual available from the authors.

### Statistical analysis

All statistical tests were performed using SPSS Software (SPSS version 28.0, IBM SPSS Statistics, RRID:SCR_016479, Armonk, NY, United States) and all descriptive results are reported as mean ± standard deviation (SD). Statistical significance was set at *p* < 0.05. One-way analysis of variance (ANOVA) was used to examine differences in baseline variables of trait physical and mental energy and fatigue. Change scores (Δ) between pre- and post-test were calculated for blood pressure, heart rate, and feelings of physical energy, physical fatigue, mental energy, and mental fatigue. The effects of group on outcome change scores and RPE were examined with one-way ANOVAs. When ANOVAs indicated significant between-groups effects, Tukey post-hoc tests were used to examine group differences. Effect sizes are presented as eta squared (η^2^). The mean differences (MD) and 95% confidence intervals (95% CI) were provided for pairwise comparisons. Within the stair walking group, correlations were calculated for floors ascended/descended with changes in physical energy, physical fatigue, mental energy, and mental fatigue.

## Results

### Trait energy and fatigue

No significant differences were found between groups for trait scores of physical energy, physical fatigue, mental energy, or mental fatigue (*p* < 0.05) and the descriptive data is provided in Table 2. The means in each group for the four trait variables were within one standard deviation of the normative means based on a random sample of 202 United States adults: physical energy (7.3 ± 2.0), physical fatigue (4.9 ± 2.3), mental energy (7.7 ± 1.8), and mental fatigue (4.5 ± 2.0).

**Table 2:** Trait energy and fatigue scores (M ± SD) by group.

	Seated laboratory	Seated stairwell	Stair walking
Trait physical energy	7.40 ± 1.35	7.72 ± 1.13	7.00 ± 1.72
Trait physical fatigue	3.60 ± 1.35	4.50 ± 1.47	4.00 ± 1.37
Trait mental energy	6.60 ± 1.43	5.94 ± 2.01	6.44 ± 1.65
Trait mental fatigue	5.90 ± 2.08	4.89 ± 1.71	4.56 ± 1.76

### Physiological measures

No significant differences were found between groups for either systolic or diastolic changes in blood pressure. The one-way ANOVA for changes in heart rate resulted in significant differences between experimental groups (*F* = 7.67, *p* = 0.001, η^2^ = 0.26). *Post-hoc* analyses revealed greater heart rate change for the stair walking group compared to both the seated stairwell control (*p* < 0.001, *MD* = 5.65, 95% CI = 1.87 to 9.42) and seated laboratory control groups (*p* < 0.001, *MD* = 5.25, 95% CI = 0.79 to 9.72). The blood pressure data are provided in Table 3. No significant associations were found for number of floors climbed with physiological variables.

**Table 3:** Outcomes (M ± SD) related to exercise intensity by group.

	Seated laboratory	Seated stairwell	Stair walking
Perceived exertion	6.00 ± 0.00	6.06 ± 0.24	9.61 ± 1.54^*^
Systolic BP (Δ)	1.33 ± 7.03	−2.55 ± 4.35	−0.02 ± 6.05
Diastolic BP (Δ)	2.77 ± 5.60	−0.74 ± 3.48	0.87 ± 6.60
Heart rate (Δ)	−1.23 ± 4.63	−1.63 ± 4.01	4.02 ± 5.25^*^

* Indicates significant difference in comparison with both seated laboratory and seated stairwell control groups (*p* < 0.01).

### Perceived exertion

As expected, the one-way ANOVA revealed significant between groups effects for RPE (*F* = 73.06, *p* < 0.001, η^2^ = 0.77). The stair walking group reported higher RPE compared to both the seated stairwell control (*p* < 0.001, *MD* = 3.56, 95% CI = 2.67 to 4.35) and seated laboratory control groups (*p* < 0.001, *MD* = 3.66, 95% CI = 2.67 to 4.55). The perceived exertion data are provided in Table 3.

### State energy and fatigue

At pre-test, no significant differences were found between groups for physical energy, mental energy, or mental fatigue. A one-way ANOVA revealed group differences in physical fatigue (*F* = 3.41, *p* = 0.04, η^2^ = 0.14). However, *post hoc* analyses demonstrated only a marginally significant difference between the seated stairwell control and stair walking groups (*p* = 0.05, *MD* = 31.17, 95% CI = −0.3 to 62.36). Across all participants, average FOE and FOF at pre-test were within one standard deviation of normative means. The means and standard deviation of the present participants and the normative means of a random sample of 202 United States adults are, respectively: physical energy (195.8 ± 40.7, 159.5 ± 56.4), physical fatigue (63.5 ± 40.6, 126.4 ± 64.7), mental energy (156.6 ± 42.4, 173.4 ± 54.5), and mental fatigue (101.1 ± 65.0, 118.0 ± 65.7).

**Figure 2:**
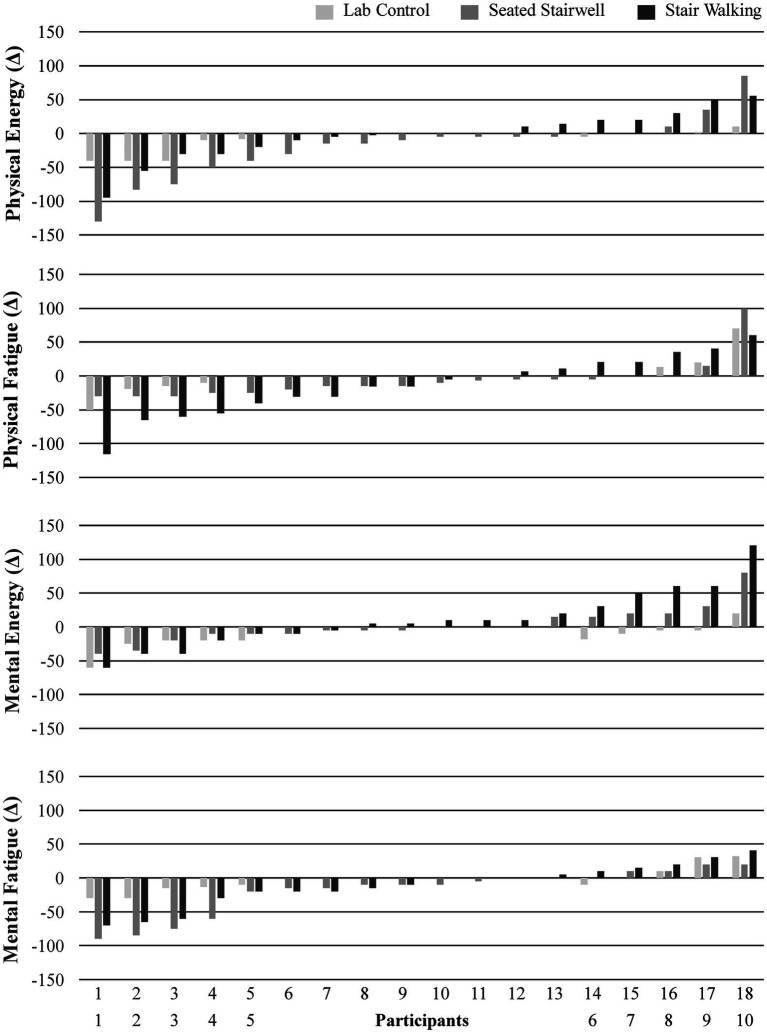
Individual changes in physical energy, physical fatigue, mental energy, and mental fatigue before and after the experimental protocol. The range of possible change is ±300. Due to the uneven group sizes, the seated laboratory control group (*n* = 10) scores are presented as the first 5 and last 5 values of the seated stairwell and stair walking groups (*n* = 18).

No significant differences were found between or within groups for state change scores of physical energy (*p* = 0.45), physical fatigue (*p* = 0.60), mental energy (*p* = 0.13), or mental fatigue (*p* = 0.46). A repeated measures ANOVA on raw scores also yielded non-significant differences. The change score data are provided in Table 4. Figure 2 displays the individual change in FOE and FOF by group. No significant associations were found for number of floors climbed with energy and fatigue variables.

**Table 4:** State energy and fatigue change scores (M ± SD) by group.

	Seated laboratory	Seated stairwell	Stair walking
State physical energy (Δ)	−13.10 ± 19.37	−18.78 ± 46.82	−2.67 ± 35.79
State physical fatigue (Δ)	0.90 ± 31.00	−6.78 ± 29.53	−13.28 ± 43.48
State mental energy (Δ)	−16.30 ± 20.12	2.22 ± 26.63	10.83 ± 42.75
State mental fatigue (Δ)	−3.60 ± 21.83	−18.61 ± 34.72	−12.20 ± 31.05

## Discussion

The primary finding was that a 4-min bout of stair walking did not improve FOE or FOF. The figures documented the large individual differences in FOE and FOF responses to the stair walking stimulus. As more data are obtained, it ultimately may be possible for individuals to choose the intervention that provides the greatest FOE and FOF benefit (e.g., some may need 20-min of walking, some may need to be outdoors and others may be able to take advantage of a micro bout of stair walking). Regardless, the present results need to be considered in the context of potential moderators of the influence of the physical activity on FOE and FOF.

One potential moderator is the intensity of the exercise. Repeatedly going up stairs (~9.6 METs) and then going down (~4.9 METs; Teh and Aziz, 2002) at a self-selected pace results in an average stair walking intensity in the vigorous range (~7.25 METs). However, heart rate in this study was elevated by only 4 bpm a few minutes after the stair walk and the mean perceived intensity was 9.6, a value closest to the verbal anchor of “very light” (9). Average perceived exertion was greater (11.4, with the closest verbal anchor described as “light”) in one stair walking study with a self-selected intensity and a duration of 10-min (Randolph and O’Connor, 2017). It is possible that the duration of stair walking in this study was insufficient to elicit the perceived exertion or blood pressure responses found in longer term studies of middle-age adults (Macfarlane et al., 2006; White et al., 2015). It also is possible that the time needed to walk back to the lab for post-test assessment lowered the post-test heart rate values. Methodological differences between our study and others are potentially useful to consider. For example, our sample was normotensive and we assessed blood pressure a few minutes after exercise while a review of the literature on post-exercise hypotension found larger effects for hypertensive samples and the magnitude of the reduction in blood pressure was larger 2-h post-exercise compared to 1-h post-exercise (Casonatto et al., 2016). Additionally, the lack of effects for blood pressure for a single short bout of stair walking are not necessarily indicative of the known improvements in hypertension for longer bout duration or sustained behavior change (Marçal et al., 2021).

Characteristics of the sample may have moderated the findings here compared to prior studies. For example, in the present study we recruited individuals who were generally healthy. In prior studies in which stair walking resulted in improved FOE, a sleep deprived sample (<45 h sleep per week) was recruited which may have contributed to exercise’s ability to increase FOE (Randolph and O’Connor, 2017). While we did not measure cardiorespiratory fitness, if it was higher in the group tested here, it may have equated to a lower relative exercise intensity while walking stairs in this study and attenuated the potential improvement in FOE. Also, while the present sample on average was within the norms for state levels of FOE and FOF at pre-test, all groups reported somewhat higher physical energy and lower physical fatigue than the estimated population mean. These baseline differences may have attenuated the potential for stair walking to positively impact physical FOE or fatigue. Likewise, the lower than normative level of pre-test mental energy in the control group may have allowed for a regression to the mean related increase in mental energy in the control group that attenuated the potential exercise effect in the ANOVA. Prior studies show that sitting typically results in no change or a worsening of FOE (Loy et al., 2013) and an increase in FOF. The seated stairwell group in the present study showed a surprising decrease in FOF that is not easily explained.

The environmental conditions may have worked against finding an increase in FOE or a decrease in FOF in the present study. Exercise performed outdoors with exposure to sunlight can augment the mood boosting effects of acute exercise (LaCaille et al., 2004) as can adding bright light during indoor exercise (O’Brien and O’Conner, 2000). The present study was conducted in an area with no natural light.

It is, of course, possible that a single bout of low-intensity stair walking has no significant effect on FOE and FOF. Our results are consistent with the study which found that six 5-min bouts of moderate-intensity walking every hour across a 6-h workday resulted in 16% higher overall FOE compared to uninterrupted sitting because there was no significant increase in FOE during the first walking bout (Bergouignan et al., 2016). So, it may be that longer duration or more intense exercise is needed to ensure improvements in FOE and FOF.

There are several strengths of this study despite the absence of meaningful change in fatigue and energy. Having a light walking group potentially contributed to the novelty in that self-selected stair walking typically appears to be of a higher intensity. External validity was increased by having participants ascend and descend the stairs compared to at least one investigation that involved only stair climbing. This mimics real world stair uses relative to studies that use a stair climbing ergometer. The findings also add to the evidence by distinguishing between both mental and physical FOE and fatigue as most studies measure fail to distinguish between these aspects or measure only FOE or FOF.

The study also has some limitations. This study is part of a broader research project and therefore used a mixed-model design rather than a completely within study design which would have reduced some error variance and increased statistical power. The study was not designed to examine dose–response relationships between FOE/FOF and either stair walking duration or intensity. The intensity was self-selected and therefore there was more variation in intensity than if the intensity was controlled. Statistical power was adequate to show a significant effect if the standardized mean effect size of 0.39 had been observed and if the correlation between measures across time was high but given the size of the observed effects and that correlations across time were not high for all the outcomes, the study was underpowered.

## Conclusion

Although a 4-min self-paced exercise bout can convey short-term physiological health benefits, a 4-min bout of self-paced indoor stair walking in a stairwell was insufficient to lower blood pressure or change subjective FOE and fatigue in a sample that exhibited better than typical FOE and fatigue at the pre-test. Future research may consider larger samples and a within-subjects design to elucidate the potential of short activity bouts to improve health.

## Data availability statement

The raw data supporting the conclusions of this article will be made available by the authors, without undue reservation.

## Ethics statement

The studies involving human participants were reviewed and approved by University of Georgia Institutional Review Board. The patients/participants provided their written informed consent to participate in this study.

## Author contributions

JG and PO’C contributed to the conception and design of the study. KC managed data collection, organized the database, and performed the statistical analyses. All authors contributed to the article and approved the submitted version.

## Funding

This project was supported in part by funds from the Workplace Health Group at the University of Georgia.

## Conflict of interest

The authors declare that the research was conducted in the absence of any commercial or financial relationships that could be construed as a potential conflict of interest.

## Publisher’s note

All claims expressed in this article are solely those of the authors and do not necessarily represent those of their affiliated organizations, or those of the publisher, the editors and the reviewers. Any product that may be evaluated in this article, or claim that may be made by its manufacturer, is not guaranteed or endorsed by the publisher.
